# Optimization of Sensing and Feedback Control for Vibration/Flutter of Rotating Disk by PZT Actuators via Air Coupled Pressure

**DOI:** 10.3390/s110303094

**Published:** 2011-03-10

**Authors:** Tianhong Yan, Xinsheng Xu, Jianqiang Han, Rongming Lin, Bingfeng Ju, Qing Li

**Affiliations:** 1 College of Mechanical & Electrical Engineering, China Jiliang University, Xueyuan Road, Xiasha High-Edu Park, Hangzhou 310018, Zhejiang, China; E-Mails: lionkingxxs@163.com (X.X.); hjqsmx@163.com (J.H.); lqing55@yahoo.cn (Q.L.); 2 State Key Laboratory of Fluid Power and Mechantronic Systems, Zhejiang University, Yuquan Road, Hangzhou 310027, Zhejiang, China; E-Mail: mbfju@sfp.zju.edu.cn; 3 State Key Lab of Digital Maufacturing Equipment and Technology, Huazhong University of Science and Technology, Wuhan 430074, Hubei, China; 4 Center for Mechanics of Micro-Systems, Nanyang Technological University, 639798, Singapore; E-Mail: mrmlin@ntu.edu.sg

**Keywords:** smart structure, control mechanism, PZT actuator, disk flutter/vibration, optimal feedback control

## Abstract

In this paper, a feedback control mechanism and its optimization for rotating disk vibration/flutter via changes of air-coupled pressure generated using piezoelectric patch actuators are studied. A thin disk rotates in an enclosure, which is equipped with a feedback control loop consisting of a micro-sensor, a signal processor, a power amplifier, and several piezoelectric (PZT) actuator patches distributed on the cover of the enclosure. The actuator patches are mounted on the inner or the outer surfaces of the enclosure to produce necessary control force required through the airflow around the disk. The control mechanism for rotating disk flutter using enclosure surfaces bonded with sensors and piezoelectric actuators is thoroughly studied through analytical simulations. The sensor output is used to determine the amount of input to the actuator for controlling the response of the disk in a closed loop configuration. The dynamic stability of the disk-enclosure system, together with the feedback control loop, is analyzed as a complex eigenvalue problem, which is solved using Galerkin’s discretization procedure. The results show that the disk flutter can be reduced effectively with proper configurations of the control gain and the phase shift through the actuations of PZT patches. The effectiveness of different feedback control methods in altering system characteristics and system response has been investigated. The control capability, in terms of control gain, phase shift, and especially the physical configuration of actuator patches, are also evaluated by calculating the complex eigenvalues and the maximum displacement produced by the actuators. To achieve a optimal control performance, sizes, positions and shapes of PZT patches used need to be optimized and such optimization has been achieved through numerical simulations.

## 1. Introduction

Rotating disks are widely used in various engineering applications, such as computer data storage systems, turbines, gyroscopes, and annular saw blades, *etc.* During the past few years, optical data recording, together with magnetic data recording, has made significant contribution to the development of information storage devices. Recent trends in optical disk drives have witnessed the transition from CD drives to high-density DVD drives, the widespread use of rewritable disk drives and the development of new types of information storage devices [1]. The demand for higher data transfer rates has led to a dramatic increase in the rotational speed of CD-ROM drives. During the last three years, the data transfer rate of CD-ROM drives has increased from 8X (8 times the transfer rate of the original CD-ROM) to 52X. DVD-ROM drives recently have repeated the same magnitude of increase in data transfer rates. With the rapid growth of data-storage density and increase of spindle motor speed in hard disk drivers (HDD), stability of rotating disks is also becoming an important issue because of its significant contribution to track mis-registration and disk drive failure. A disk rotating at high speed can lose its stability due to the coupling of the disk structure with its surrounding airflow in such a way that it keeps absorbing mechanical energy from the airflow to quickly build its vibration amplitude until failure sets in. This is called aeroelastic instability, or flutter. The vibration of a rotating disk coupled with surrounding airflow has been of interest to many researchers in past decades. A number of scientists have studied the aeroelastic stability (stability to self-excited vibrations) of spinning disks and obtained interesting results [2–5].

As the rotational speed increases, the backward natural frequency of a rotating disk decreases and vanishes at a certain critical speed. At this critical speed, a stationary transverse force provokes resonances and causes buckling instability in the rotating disk. A single backward traveling wave is excited at a certain speed above the critical speed. However, one of the important issues to be considered for higher rotational speeds is aerodynamic flutter. The self-excited vibration can be induced by the aerodynamic forces around a rotating disk. Since Lennemann [6], who studied aerodynamic effect on rotating disk files, many researchers have studied aerodynamically induced disk vibrations. Hosaka and Crandall [7] and Huang and Mote [8] have investigated the vibration stability of a flexible disk spinning very close to a rigid wall. In those papers, classical hydrodynamic lubrication theory was applied assuming that Reynold’s number of the fluid flowing in the gap is very small. Recently, Kim *et al.* [9] have predicted that the flutter speed of a hard disk drive can be about 35,000 rpm. Lee and Kim [10] also observed aero-induced flutter in optical disks. A modal extraction method to predict flutter of rotating disks was studied by the estimation of nonconservative aerodynamic pressures [11]. They suggested a similar empirical model to predict flutter of a disk rotating in a fluid where the relationship between the speed of the disk and the damping force was established according to some experimentally determined parameters. When a disk spins in open air without an enclosure, aerodynamic flutter occurs at lower speeds than a disk spinning close to a rigid wall. It is because the nonsymmetric stiffness, generated by the radial component of the airflow in the lubrication film, stiffens the disk. Yasuda *et al.* [12] proposed that the aerodynamic force on a disk rotating in an infinite medium can be written in terms of the so-called lift and damping forces whose models can be characterized as rotating damping. They assumed that the ratio between the lift and damping force was proportional to rotational speed. Similar to the analysis of a disk rotating close to a rigid wall, they showed that flutter of a single backward traveling wave could appear at certain specific rotational speeds. Renshaw [13] studied the stability of a flexible floppy disk rotating very close to a rigid wall. The analyses were based on the classical hydrodynamic lubrication theory to describe the mechanism of thin viscous flow in the gap coupling to the disk. D’Angelo and Mote [14] and Renshaw *et al.* [15] experimentally investigated the vibration of a thin disk rotating in atmospheres of different densities and observed disk flutter and flutter modes. The research activities in disk flutter have recently been directly applied to hard disk drives. Imai *et al.* [16] and Imai [17] experimentally investigated the mechanism of disk flutter excitation in hard disk drivers and suggested a technique of deceasing disk-to-shroud spacing to reduce amplitudes of disk flutter. Heo *et al.* [18] presented another control method, which used a better aerodynamic design of the shroud contour on the base casting to reduce the disk flutter. Bittner *et al.* [19] proposed an acoustically tuned-mass damper to suppress disk flutter through an air bearing plate on top of the disk. By applying a squeeze film damping to a commercially available HDD, Deeyiengyang *et al.* [20] conducted experiments to study the effect of clearances between squeeze film and disk surface on suppressing vibration of spinning disk/spindle systems. All these methods mentioned are passive control techniques, which either modify the configuration of the disk drive casings and hence the aerodynamic characteristics, or increase the dynamic damping of the disk. Yan *et al.* [21] had preliminarily studied active feedback control method to decrease vibration/flutter of rotating disks of HDDs via distributed actuators. Considering the space limit, only self-sensing distributed actuators (rectangular PZT) had been studied experimentally. Huang *et al.* [22] had investigated theoretically and deeply the active control method to suppress the rotating disk flutter, in which the disk vibration signals were detected and processed to generate pressure perturbations inside the disk enclosure in such way that the original coupling would be altered, leading to the suppression of disk flutter. This was then also studied experimentally through a prototype disk system setup—a disk rotating between two fixed plates—by Huang *et al.* [23]. The feedback control systems generally require sensors and actuators, which are relatively easy to implement in laboratory experiments. In real hard disk drives however, sensors might present a problem when implementing feedback control techniques practically limited space. D’Angelo *et al.* [24] theoretically predicted flutter instability and frequency lock-on phenomenon of a rotating steel disk. Aerodynamic effect on natural frequencies and flutter speeds of rotating disks are experimentally studied using a vacuum chamber and optical disks. Experimental results demonstrate that the natural frequencies of a rotating disk in vacuum are larger than those obtained in ambient pressure in pre-flutter regions. Furthermore, the backward natural frequency of the disk is observed to be equal to that of the case without aerodynamic effect at flutter onset speed.

In this paper, the ratio, which is related to the magnitudes of lift and damping forces, is assumed to be proportional to rotational speed, and it has been shown that disk flutter could possibly arise at certain rotational speeds. The systematic studies on piezoelectric patches as actuators their performance of control under various arrangements of a number of actuator patches. The thin piezoelectric patches are attached on the surface of cover plate of the enclosure and vibrations of these actuator patches generate the required control forces through disturbed acoustic field in the disk-enclosure system. Dynamic stability of the disk-enclosure system, together with the feedback control loop, is analyzed as a complex eigenvalue problem which is solved using Galerkin’s discretization procedure. By taking into account the air-coupling and control force on the rotating disk, its dynamic stability is analyzed using Galerkin’s method. It has been demonstrated that, with a proper combination of control gain and phase shift, disk flutter can be suppressed effectively using piezoelectric actuator patches. Further, the control capability of different configurations, in terms of size, location, and shape of different number of actuator patches distributed on the surfaces of cover plate of the enclosure, have also been studied numerically. Some optimal configurations in terms of size and location arrangement of the actuator patches have been obtained, including both passive and active actuator using for HDDs.

The remaining sections of the paper are organized as follows. Section 2 develops the theoretical modeling and equations of motion, the analytical method is presented in Section 3, while Section 4 illustrates and discusses the simulation results. The final two sections include the numerical results for different control configurations of PZT patches and a conclusion.

## 2. Theoretical Modeling and Equations of Motion

**Figure 1.**
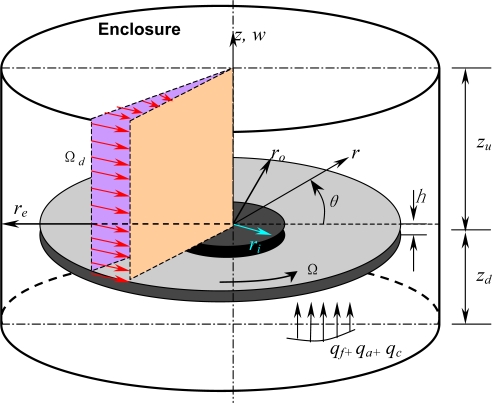
A schematic diagram of a rotating disk in the presence of fluid media in a cylindrical enclosure.

Consider an annular disk with a uniform thickness *h* and an outer radius *r_o_*, clamped at the center with an inner radius *r_i_* in a cylindrical enclosure, as shown in Figure 1. The disk rotates about its vertical axis at a constant angular speed Ω. The material parameters of the disk which are Young’s modulus, Poisson’s ratio, and density are given as *E*, *ν*, and *ρ_d_*, respectively. The enclosure has radius *r_e_* and height *z_u_* + *z_d_* = 2*z_e_*, and the density of air in the enclosure is *ρ_a_*. The rotating disk undergoes small magnitude of transverse motions modeled based on linear Kirchhoff plate theory with in-plane stresses. The governing equation in terms of transverse displacement *w̄*(*r̄*, *θ*, *t̄*), with respect to the space-fixed coordinates (*r̄*, *θ*, *t̄*), can be written in a nondimensional form as follows [9,11,13,15,22,25]:

(1) $$\left(\frac{\partial^{2}w}{{\partial t}^{2}}+2\frac{\partial^{2}w}{\partial t\partial \theta }+\frac{\partial^{2}w}{{\partial \theta }^{2}}\right)+{\mu \nabla }^{4}w-\left[\frac{1}{r}\frac{\partial }{\partial r}\left({r\sigma }_{r}\frac{\partial w}{\partial r}\right)+\frac{1}{r^{2}}\frac{\partial }{\partial \theta }\left(\sigma_{\theta }\frac{\partial w}{\partial \theta }\right)\right]=q\left(r,\;\;\theta ,\;\;t\right)$$

where $w=\frac{\bar{w}}{h}$, $r=\frac{\bar{r}}{r_{o}}$, $z=\frac{\bar{z}}{r_{o}}$, *t* = Ω*t̄*, $\kappa =\frac{r_{i}}{r_{o}}$, $\sigma_{r}=\frac{{\bar{\sigma }}_{r}}{\rho_{d}\;r_{o}^{2}\Omega^{2}}$, $\sigma_{\theta }=\frac{{\bar{\sigma }}_{\theta }}{\rho_{d}\;r_{o}^{2}\Omega^{2}}$, $q=\frac{\bar{q}}{\rho_{d}\;h^{2}\Omega^{2}}$. $\mu =D/\rho_{d}\;r_{o}^{4}\Omega^{2}h$ is the ratio between disk bending stiffness and the stiffness derived from centrifugal body forces due to rotation. *D* = *Eh*^3^/12(1 – *ν*^2^) is the bending rigidity of the disk, and $\nabla^{4}={\left(\frac{\partial^{2}}{{\partial r}^{2}}+\frac{\partial }{r\partial r}+\frac{\partial^{2}}{r^{2}{\partial \theta }^{2}}\right)}^{2}$ is the biharmonic differential operator. Here, *q*(*r*, *θ*, *t*) represents the transverse loadings acting on the rotating disk, which includes aerodynamic force *q_f_* (*r*, *θ*, *t*), acoustic force *q_a_* (*r*, *θ*, *t*), and control force *q_a_* (*r*, *θ*, *t*). Here *σ_r_* and *σ_θ_* are the radial and hoop membrane stresses which satisfy the generalized plane-stress equations of linear elasticity with centrifugal body forces [26] and are expressed as follows:

$$\begin{array}{c} {\bar{\sigma }}_{r}=\rho_{d}\;r_{o}^{2}\Omega^{2}\left[b_{0}{\left(\frac{r_{o}}{r}\right)}^{2}+b_{1}-\frac{3+v}{8}{\left(\frac{r}{r_{o}}\right)}^{2}\right]\\ {\bar{\sigma }}_{\theta }=\rho_{d}\;r_{o}^{2}\Omega^{2}\left[-b_{0}{\left(\frac{r_{o}}{r}\right)}^{2}+b_{1}-\frac{1+3v}{8}{\left(\frac{r}{r_{o}}\right)}^{2}\right]\\ b_{0}=\frac{\left(1-v\right){\left(r_{i}/r_{o}\right)}^{2}\left[\left(3+v\right)-\left(1+v\right)\left({\left(r_{i}/r_{o}\right)}^{2}\right)\right]}{8\left[\left(1+v\right)+\left(1-v\right){\left(r_{i}/r_{o}\right)}^{2}\right]}\\ b_{1}=\frac{\left(1-v\right)\left[\left(3+v\right)+\left(1-v\right)\left({\left(r_{i}/r_{o}\right)}^{4}\right)\right]}{8\left[\left(1+v\right)+\left(1-v\right){\left(r_{i}/r_{o}\right)}^{2}\right]} \end{array}$$

There are four radial boundary conditions associated with Equation (1). At the clamped edge *r* = *r_i_* the transverse displacement and its rotation (slope) are zeros:

(2a) w=0

(2b) ∂w/∂r=0

and at outer edge *r* = *r_o_*, the radial bending moment and the shear force reaction are zeros:

(3a) $$w_{,\;\mathrm{rr}}+v\left(w_{,\;r}/r+w_{,\;\theta \theta }/r^{2}\right)=0$$

(3b) $${\left(\nabla^{2}w\right)}_{,\;\mathrm{rr}}+\left(1-v\right)/r^{2}{\left(w_{,\;r}-w/r\right)}_{,\;\theta \theta }=0$$

as well as additional condition of circumferential periodicity, *w*(*r*, *θ*, *t*) = *w*(*r*, *θ* + 2*π*, *t*).

### 2.1. Aerodynamic Force and Acoustic Force

The empirical model of aerodynamic force, proposed by Kim *et al.* [9] and Hansen *et al.* [11] is employed in this study for the aerodynamic force. This hydrodynamic model, based on the rigorous Navier–Stokes equations, for description of aerodynamic force arising from airflow of disk rotation in an enclosure is highly complicated and needs to be simplified somehow in order to provide an analytical design method. Here, the model adopted is based on a simple mechanism that takes the aerodynamic loading exerted on the disk as a distributed viscous damping force rotating relative to the disk. It has some inherent benefits of complex system analysis but it avoids the solution of the complete Navier–Stokes equations, yet it gives good predictions on the rotating disk flutter qualitatively [22,23]. The model of aerodynamic force proposed by Kim *et al.* [9] is employed here, it has the form:

(4) $$q_{f}\;\left(r,\;\;\theta ,\;\;t\right)=-C\left[\frac{\partial w}{\partial t}+\left(1-\frac{\Omega_{d}}{\Omega }\right)\frac{\partial w}{\partial \theta }\right]$$

where *C* is a damping coefficient depending on the viscosity of the fluid, the rotational speed of the disk and the geometrical parameters of the enclosure, and Ω*_d_* is the rotational speed of the distributed viscous damping force relative to the disk. Both *C* and Ω*_d_* should be determined by experiments.

The acoustic force *q_a_* (*r*, *θ*, *t*) on the disk can be calculated through the pressure difference between the upper and the lower surfaces of the disk, and can be written as:

(5) $$q_{a}\;\left(r,\;\;\theta ,\;\;t\right)=\Lambda \left[{\frac{{\partial \phi }_{a}\;\left(r,\;\;\theta ,\;\;z,\;\;t\right)}{\partial t}|}_{z=0^{+}}-{\frac{{\partial \phi }_{a}\;\left(r,\;\;\theta ,\;\;z,\;\;t\right)}{\partial t}|}_{z=0^{-}}\right]$$

where *ϕ_a_* is the acoustic velocity potential and *Λ* = *ρ_a_r*_0_/*ρ_d_h*. The governing equation for the acoustic field in the enclosure is expressed by:

(6) $$\nabla^{2}\phi_{a}=M^{2}\frac{\partial^{2}\phi_{a}}{{\partial t}^{2}}$$

where $\nabla^{2}=\frac{\partial^{2}}{{\partial r}^{2}}+\frac{\partial }{r\partial r}+\frac{\partial^{2}}{r^{2}{\partial \theta }^{2}}+\frac{\partial^{2}}{{\partial z}^{2}}$ is the space Laplacian operator, and *M* = *r*_0_ Ω/*a* in which *a* is the speed of the sound in the enclosure. The acoustic velocities on the enclosure walls must be zero and hence the boundary conditions for *ϕ_a_* are:

(7a) $${\frac{{\partial \phi }_{a}}{\partial r}|}_{r=r_{e}}=0$$

(7b) $${\frac{{\partial \phi }_{a}}{\partial z}|}_{z={+z}_{u}}=0,\;\;{\frac{{\partial \phi }_{a}}{\partial z}|}_{z={-z}_{d}}=0$$

where *r_e_* = *r̄_e_*/*r_o_*, and *z_u_* = *z̄_u_*/*r_o_*, *z_d_* = *z̄_d_*/*r_o_*.

In addition, on the surfaces of the disk, the acoustic velocities should match the corresponding disk vibration velocities, and at the clearance between the disk rim and the enclosure, *ϕ_a_* = 0 for asymmetric acoustic field [15]. As a result, we have the following conditions:

(8a) $${\frac{{\partial \phi }_{a}}{\partial z}|}_{z=0}=\{\begin{array}{ll} 0 & \left(0\leq r\leq \kappa \right)\\ \frac{\partial w}{\partial t} & \left(\kappa \leq r\leq 1\right) \end{array}$$

(8b) $${\phi_{a}|}_{z=0}=0,\;\left(1<r\leq r_{e}\right)$$

### 2.2. Control Force Induced by PZT Actuator Patches

**Figure 2.**
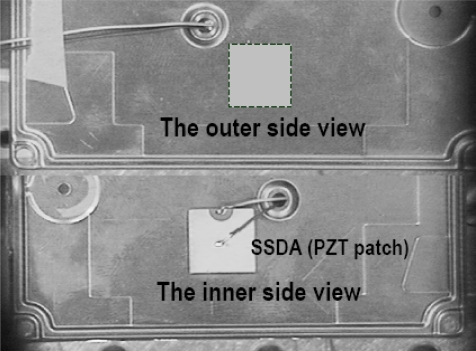
The Feedback Control of Disk Vibration/Flutter via DSSA [21].

In this section, an acoustic feedback control is introduced into the disk-enclosure system, as presented in [21], as shown in Figure 2, the feedback control force is generated by the PZT actuators located on the cover of cylindrical enclosure, whose shapes are similar to passive squeeze plates, but they are attached on the surfaces of the enclosure plate.

A sensor is placed in the enclosure to pick up the vibration of the disk at point (*r_s_*, *θ_s_*). The signal is then amplified and phase-shifted to become a driving signal to drive the actuator. The actuator will then generate a surface vibration on the upper surface of the enclosure. This surface vibration is therefore dependent on the control gain *G*, the phase shift *σ*, the disk vibration at sensor location, and the actuator distribution *A*(*r*, *θ*) on the upper surface, as shown in Figure 4. In general, *A*(*r*, *θ*) can be written as [22]:

(9) $$A\left(r,\;\theta \right)=\sum_{n=-\infty }^{+\infty }a_{n}\left(r\right)e^{\mathrm{in}\theta }$$

**Figure 3.**
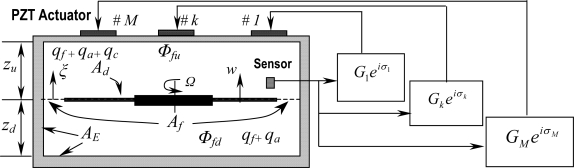
Schematic diagram of feedback control with multi-actuators.

In the feedback control, we only take the *n* -th mode and consider a simple case that *a_n_* (*r*) is a constant in the actuator region *r*_1_ ≤ *r* ≤ *r*_2_ with *θ*_1_ ≤ *θ* ≤ *θ*_2_. Due to the motion of the actuator, a controlled acoustic field will be generated in the enclosure, which is denoted as *ϕ_c_*. On the actuating portion, the acoustic velocity induced by *ϕ_c_* shall be the same as the actuator velocity, which can be written as *Ge^iσ^ e^inθ^* ∂*w*(*r_s_*, *θ_s_* *t*)/∂*t*, and on other parts of the upper surface, the acoustic velocity is zero. If there exist more than one actuator patches located at different positions on upper surface, denoted by an integer *j* = 1,2, ⋯, *J*, as shown in Figure 3, the distribution function of each actuator is *A_j_* (*r_j_*, *θ_j_*). On the *j* th actuating portion, the acoustic velocity induced by *ϕ_c_* equals to the *j* th actuator velocity which can be written as *G_j_e*^*iσ_j_*^ *e*^*inθ_j_*^ ∂*w*(*r_s_*, *θ_s_t*)/∂*t*. Since the gap between the disk rim and the sidewall of the enclosure is normally very small, we can assume that this gap is negligible and as a result, the acoustic velocity induced by *ϕ_c_* on the disk becomes zero (the acoustic field induced by the disk vibration has been considered in *ϕ_a_*). The governing equation and the boundary conditions for the controlled acoustic field *ϕ_c_* are therefore written as:

(10) $$\nabla^{2}\phi_{c}=M^{2}\partial^{2}\phi_{c}/{\partial t}^{2}$$

(11) $${\frac{{\partial \phi }_{c}}{\partial z}|}_{z=z_{u}}=\{\begin{array}{ll} G_{k}e^{{i\sigma }_{k}}e^{{\mathrm{in}\theta }_{k}}\frac{\partial w\left(r_{s},\;\theta_{s},\;t\right)}{\partial t} & \left(r_{1k}\leq r_{k}\leq r_{2k},\;\theta_{1k}\leq \theta_{k}\leq \theta_{2k},\;k=1,\;2,\;\cdots ,\;M\right)\\ 0 & \left(r,\;\theta \right)\notin \left(A_{1}\cup A_{2}\cup \cdots \cup A_{k}\cup \cdots \cup A_{M}\right) \end{array}$$

(12a) $${{\partial \phi }_{c}/\partial z|}_{z=0}=0$$

(12b) $${{\partial \phi }_{c}/\partial r|}_{r=r_{e}}=0$$

The controlled acoustic field *ϕ_c_* will generate an additional force on the disk surface, denoted by *q*_c_, which can be calculated by:

(13) $${q_{c}=\Lambda \frac{{\partial \phi }_{c}\left(r,\;\theta ,\;z,\;t\right)}{\partial t}|}_{z=0^{+}}$$

This control force will be added to the right-hand side of Equation (11) and the vibration of the disk-enclosure system is therefore under control.

## 3. Analytical Method

In order to solve air-coupled disk vibration equations, Equation (1) will be solved under all the forces and the required boundary conditions. All governing equations for the rotating disk and acoustic fields should be solved simultaneously. To achieve this, approximate method used by previous investigators [7,15,22,25] was employed here. Similarly, separable forms for the transverse displacement *w*(*r*, *θ*, *z*, *t*), and the acoustic velocity potentials *ϕ_a_* (*r*, *θ*, *z*, *t*) and *ϕ_c_* (*r*, *θ*, *z*, *t*), are assumed:

(14a) $$w\left(r,\;\theta ,\;z,\;t\right)=R\left(r\right)e^{i\left(n\theta +\lambda T\right)}$$

(14b) $$\phi_{a}\left(r,\;\theta ,\;z,\;t\right)=\psi_{a}\left(r,\;z\right)e^{i\left(n\theta +\lambda T\right)}$$

(14c) $$\phi_{c}\left(r,\;\theta ,\;z,\;t\right)=\psi_{c}\left(r,\;z\right)e^{i\left(n\theta +\lambda T\right)}$$

where *R*(*t*), *ψ_a_* (*r*, *z*), and *ψ_c_* (*r*, *z*) are unknown functions to be determined. *λ* is the eigenvalue whose real part determines disk vibration frequency and imaginary part indicates the stability of the system. *R*(*t*) is obtained by Galerkin’s method in the following form:

(15) $$w\left(r,\;\theta ,\;z,\;t\right)=\sum_{m=0}^{\infty }c_{m}R_{\mathrm{mn}}\left(r\right)e^{i\left(n\theta +\lambda T\right)}$$

where *m* and *n* represent the number of nodal circles and number of nodal diameters of the vibration mode (*m*, *n*), and *c_m_* are coefficients. In numerical simulation, the infinite series in Equation (15) will be truncated at *m* = *M*_0_ within allowable accuracy and a power series is used to approximate *R_mn_* [27]:

(16) $$R_{\mathrm{mn}}\left(r\right)=r^{m}+r^{m+1}+E_{\mathrm{mn}}^{\left(1\right)}r^{m+2}+E_{\mathrm{mn}}^{\left(2\right)}r^{m+3}+r^{m+4}+E_{\mathrm{mn}}^{\left(3\right)}r^{m+5}+E_{\mathrm{mn}}^{\left(4\right)}r^{m+6}$$

where $E_{\mathrm{mn}}^{\left(i\right)}$, (*i* = 1, 2, 3, 4) are constants to be determined such that all the boundary conditions of the disk are satisfied. The acoustic velocity potentials *ϕ_a_* and *ϕ_c_* are solved according to the boundary conditions and have the following form:

(17) $$\phi_{a}\left(r,\;\theta ,\;z,\;t\right)=\sum_{i=1}^{\infty }d_{k}^{a}\text{cosh}\left[\alpha_{k}\left(z_{e}-z\right)\right]J_{n}\left(\xi_{k}r\right)e^{i\left(n\theta +\lambda T\right)}$$

(18) $$\phi_{c}\left(r,\;\theta ,\;z,\;t\right)=\sum_{i=1}^{\infty }d_{k}^{c}\text{cosh}\left(\alpha_{k}z\right)J_{n}\left(\xi_{k}r\right)e^{i\left(n\theta +\lambda T\right)}$$

where *J_n_* (*ξ_k_ r*) is a Bessel function of *n*th order, *ξ_k_* is determined by the roots of *J*′*n* (*ξ_k_* *r*) = *0* (*k* = *1*, *2*, ⋯, ∞), which represents the boundary conditions at the sidewall of the enclosure, $\alpha_{k}=\sqrt{\xi_{k}^{2}-{\left(M\lambda \right)}^{2}}$, $d_{k}^{a}$ and $d_{k}^{c}$ will be determined, respectively, by matching condition (8) at *z* = 0 and the boundary condition (12) at *r* = *r_e_*. Owing to the couplings between disturbed acoustic fields and disk vibrations, $d_{k}^{a}$ and $d_{k}^{c}$ are functions of coefficients *c_m_*. That is, $d_{k}^{a}=d_{k}^{a}(c_{m})$ and $d_{k}^{c}=d_{k}^{c}(c_{m})$.

For two arbitrary complex-valued functions *a*(*r*, *θ*) and *b*(*r*, *θ*) which are defined in domain {*k* ≤ *r* ≤ 1, 0 ≤ *θ* ≤ 2*π*}, we define an inner product as follows:

(19) $$<a\left(r,\;\theta \right),\;b\left(r,\;\theta \right)>=\int_{0}^{2\pi }\int_{k}^{1}a\left(r,\;\theta \right)b^{*}\left(r,\;\theta \right)r\;\mathrm{dr}\;d\theta$$

where the superscript asterisk denotes complex conjugate. By substituting Equations (15), (17) and (18) into the governing Equation (1) of the disk, and calculating the inner product with *R_ln_e*^*i*(*nθ*+*λt*)^, (*l* = *0*, *1*, *2*, ⋯, *M_0_*), one obtains a matrix equation for the coefficients *c_m_* following Galerkin’s method:

(20) $$\left\{\left[B\right]+\left[P^{f}\right]+\left[P^{a}\right]+\left[P^{c}\right]\right\}\left[c\right]=\left[0\right]$$

where $[c]=[{\begin{array}{llll} c_{0} & c_{1} & \cdots & c_{M_{0}} \end{array}]}^{T}$, [*B*] is a (*M*_0_ + 1)×(*M*_0_ + 1) square matrix associated with free vibration of the rotating disk without any aerodynamic loading, [*P^f^*] is a (*M*_0_ + 1)×(*M*_0_ + 1) square matrix associated with aerodynamic force due to disk rotation, [*P^a^*] and [*P^c^*] are evaluated according to Appendix A of [22]. The elements for [*B*] and [*P^f^*] are given as follows:

(21) $$B_{\mathrm{ml}}=2\pi \int_{k}^{1}[{\left(\lambda +n\right)}^{2}R_{\mathrm{mn}}\left(r\right)-\mu \nabla_{n}^{4}R_{\mathrm{mn}}\left(r\right)+\frac{1}{r}\left(r\sigma_{r}\frac{dR_{\mathrm{mn}}}{\mathrm{dr}}\right)-\frac{n^{2}}{r^{2}}\sigma_{\theta }R_{\mathrm{mn}}\left(r\right)]R_{\ln}\left(r\right)r\;\mathrm{dr}$$

(22) $$P_{\mathrm{ml}}^{f}=-2\pi \int_{k}^{1}C_{i}\left[\lambda +\left(1-\frac{\Omega_{d}}{\Omega }\right)n\right]R_{\mathrm{mn}}\left(r\right)R_{\ln}\left(r\right)r\;\mathrm{dr}$$

where $\nabla_{n}^{4}={\left(\frac{d^{2}}{{\mathrm{dr}}^{2}}+\frac{d}{\mathrm{rdr}}-\frac{n^{2}}{r^{2}}\right)}^{2}$. In Equation (20), [*P^f^*] and [*P^c^*] are additional terms which were not included in the model of Renshaw *et al.* [15]. The condition for the existence of non-trivial solutions for Equation (20) leads to a characteristic equation:

(23) $$\text{det}\left\{\left[B\right]+\left[P^{f}\right]+\left[P^{a}\right]+\left[P^{c}\right]\right\}=0$$

from which the eigenvalue *λ* is obtained from the roots. These roots come in (*M*_0_ + 1) pairs and generate (*M*_0_ + 1) pairs of eigenvalues for a fixed nodal diameter *n*. Each pair of eigenvalues is denoted by *λ^FTW^* and *λ^BTW^* for Forward Traveling Wave (FTW) and Backward Traveling Wave (BTW) along and against the rotation direction of the disk [9,11].

The real parts of the eigenvalues, *Re* (*λ*), are related to disk vibration mode frequencies while the imaginary parts, *Im*(*λ*), are related to the ‘damping’ of a disk vibration with a condition of *Im*(*λ*) > 0 indicates an unstable vibration or flutter. If a disk rotates in vacuum, all its eigenvalues are just real numbers and the system is therefore stable. If a disk rotates in air without implementing feedback control, the eigenvalues will be complex numbers and *Im*(*λ*) may be negative for some modes, *i.e.*, flutter may occur in some of the modes. All these cases are demonstrated and discussed next.

## 4. Simulation Results and Discussions

**Figure 4.**
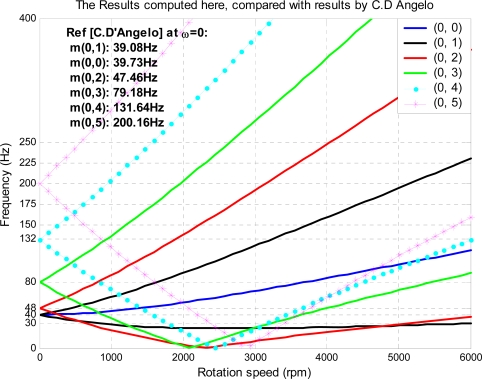
Mode frequency *versus* rotational speed of disk platter without air coupling.

Case studies are conducted in this section to show that flutter may occur for some modes and that it is possible to suppress flutter by the proposed feedback control technique. First, the disk used in this simulation is the same as the one used by D’Angelo and Mote [14,24] so that we can compare results for mode verification and updating. Details of the disk investigated can be found in references [14,24]. The critical speed for mode (0, 3) in present study has been found to be 2,081 rpm, which is comparable to the measured value of 2,078 rpm reported in [14,24] with a percentage error of less than 3.0%, as shown in Figure 4.

Second, the hard disk platter is investigated with geometric and material parameters listed in Table 1. During the simulations, the enclosure dimensions are assumed to be fixed at *z_e_* = 0.5* *r_o_* and *r_e_* = 1.2* *r_o_* and the sensor is assumed to be located at (*r_s_*, *θ_s_*) = (0.9, 0) and *r_a_* = 0.8.

**Figure 5.**
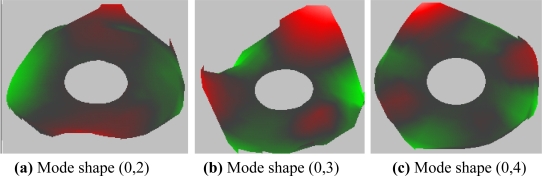
Measured Modeshapes of Modes (0,2), (0,3) and (0,4) of Disk Platter.

The calculated mode frequencies for the rotational disk are compared with the measured results by Imai [17], and the results for some modes are listed in Table 2 (with rotating speed Ω = 5,400 rpm). The errors are less than 5% for most of modes, except 6.62% for mode (0, 0). For disk without rotating, the measured typical mode shapes (0,2), (0,3), and (0,4) are shown in Figure 5. After algorithm verification, the studies of HDD with feedback control are shown next.

**Figure 6.**
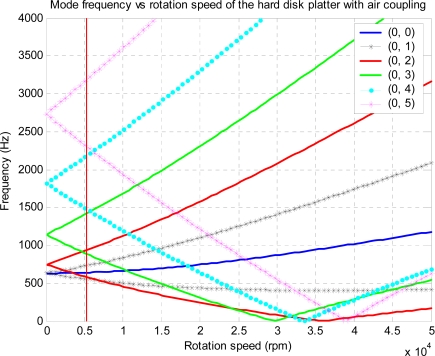
Mode frequency *versus* rotational speed of disk platter in the enclosure with air coupling.

**Figure 7.**
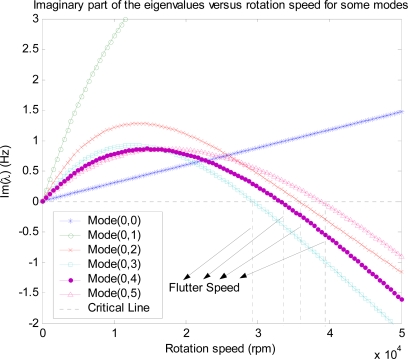
Imaginary part of the eigenvalues *versus* rotational speed for modes (0,0)∼(0,5) of the disk in the enclosure with air-coupling.

The aerodynamic loading described by Equation (4) is an empirical model in which nondimensional parameter *C* and speed ratio Ω*_d_*/Ω in Equation (11) should be determined according to experimental data. Our simulation indicates that the flutter speed is not sensitive to the damping coefficient, so that we set *C* = 0.01 by considering that the aerodynamic loading is a kind of ‘damping’ before onset of flutter and it should be light compared to the material damping of the disk, which is in order of 0.01–0.1 [28]. An experiment has been conducted [22] to measure the flutter speeds of two different disks and found that the speed ratio Ω*_d_*/Ω in the model should be in a range of 0.8–0.86 in order to for the predicted flutter speeds to agree with measured values. In the present study, we set Ω*_d_*/Ω = 0.8 and for this value, we find that the predicted flutter mode and speed are very close to the observed values. The real parts and the imaginary parts of the eigenvalues have been converted into mode frequencies and mode dampings, respectively, and the results *versus* rotational speed are plotted in Figure 6. Consisting of FTWs and BTWs. The imaginary parts of the eigenvalues, which are shown in Figure 7, are not zero due to aerodynamic loadings. It is seen that damping for BWT modes (0, 3), (0, 4) and (0, 5) are initially positive, but become negative as rotational speed increases, indicating that the rotating disk is unstable or flutter occurs above these speeds, both *ϕ_a_* and *ϕ_c_* combined are the source of the instability. The speed at which damping changes from positive to negative is denoted as the flutter speed. Figure 7 shows that flutter occurs first in mode (0, 3), which agrees with observation made by Imai [17]. The flutter speed corresponding to (0, 3) mode is found to be 29,350 rpm for the disk under study. For modes (0, 4), (0, 2), and (0.5), flutter occur at 23,314 rpm, 35,920 rpm, and 38,925 rpm, respectively. It should be pointed out that the accuracy of flutter prediction depends on the model of the aerodynamic loading on the disk, which is another research topic involving aerodynamics of flow around the rotating disk and the disk-air coupling.

**Figure 8.**
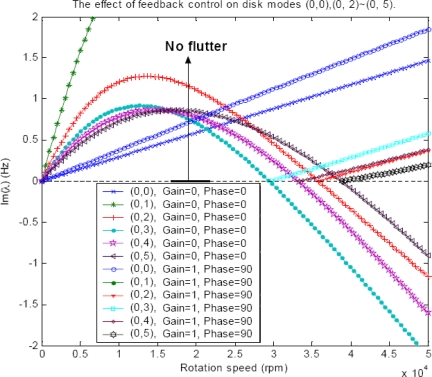
The effect of feedback control on *Im* (*λ*) with Gain = 1.0 and Phase (shift) = 90° for disk modes (0, 2)∼(0, 5) *versus* rotational speed.

The present study focuses on whether flutter can be suppressed by the feedback control technique, and this will be discussed next. If the feedback control is actuated by adding the control term [*P^c^*] in Equation (30), and the control performance is evaluated based on the effect of the control on the imaginary parts of the eigenvalues. It shows that the damping curves are lifted up by the control for modes (0.2), (0, 3), (0, 4) and (0, 5) when the phase shift is set at *σ* = π/2, as shown in Figure 8. In other words, the flutter speeds for these modes have been increased when feedback control is introduced, and the stability of the rotating disk is therefore improved. How much stability can be improved depends on the control gain. At the correct phase shift (e.g., *σ* = π/2), the greater the gain is, the more stable the disk will be. For the same configuration of distributed PZT actuator, the performance of the control scheme is dependent mainly on the gain. It indicates that the feedback control is robust when optimal parameters are chosen including the sizes and locations of the distributed PZT actuators.

It may be interesting and beneficial to mention the control mechanism in feedback control of disk flutter. In fact, the aerodynamics involved in the coupling between the air and rotating disk are quite complicated, which is the reason why there are no theoretical models for the aerodynamic loading term, but only empirical ones. However, since flutter is a kind of aeroelastic instability in which aerodynamic energy is supplied to the disk structure through the coupling at a rate faster than it can dissipate, it is reasonable to suggest that the feedback control in this case acts to weaken the air-disk coupling so as to prevent the energy from being supplied into the disk structure. It would be helpful to view the disk with feedback control as a complete dynamical system which is more stable than the original one without control. In engineering practice, it is important to give a guidance as how to obtain an optimal design when using distributed PZT actuators for passive and active flutter control. The characteristics and performance of different configuration of the PZT actuators have been investigated numerically and details will be discussed in the next section.

## 5. Numerical Studies of Different Feedback Control Schemes

### 5.1. Control Force Induced by Passive Squeezing Patches

**Figure 9.**
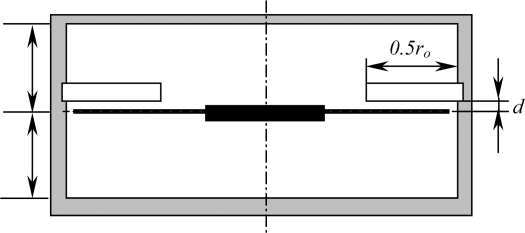
Passive Squeezing Plates for Rotating Disk Vibration/Flutter Control.

**Figure 10.**
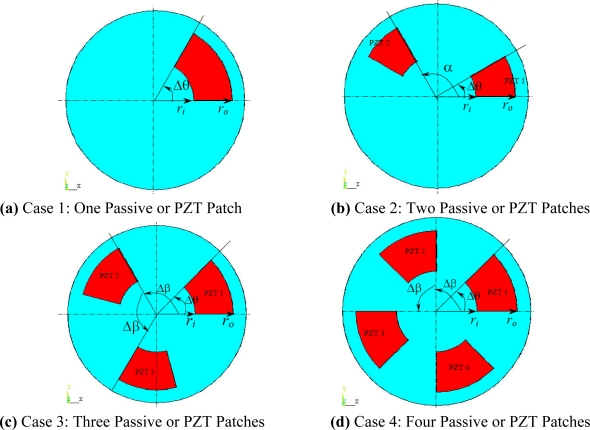
Passive Squeezing Plates for Vibration and Flutter Control (Top View).

The flutter/vibration of rotating disk can be decreased also by passive squeezing patches [20], as shown in Figure 9, but the configurations of passive patches in terms of number, dimensions and layout have major effect of suppression effectiveness. With the disk rotating at 5,000 rpm, four different case studies are designed and simulated, as shown in Figure 10. The maximum pressure and fluid velocity induced by different passive plates are numerically studied, summarized in Table 3. The contour plots of pressure and velocity generated by passive squeezing patches are shown in Table 4. The generated pressure is maximal when the passive squeezing plate is as near to the disk as possible.

### 5.2. Control Forces Induced by Different Active Squeezing Patches on Enclosure

In order to obtain approximate optimal parameters for each configuration, only the maximum displacement that is generated by each PZT actuator with different angles is compared here, with the inner radius *r_i_* and outer radius *r_o_* being fixed as 15 mm and 30 mm, respectively. The different sizes and shapes of active PZT actuator, as shown in Figure 10, are also simulated under 10 volts.

Detailed simulation results are shown in Table 5. In the case of one single actuator patch, a better control for the first three flutter modes can be achieved when the PZT sector is the 360 degree in center of disk cover. For flutter mode of a disk in real operating conditions to be control, the corresponding configuration needs to be selected.

## 6. Conclusions

Feedback control mechanism using PZT patches via air-coupled pressure to suppress flutter of rotating disks in an air-filled enclosure has been studied in this paper. The stability of the rotating disk enclosure system, together with the feedback control, has been studied by calculating and examining the eigenvalues of the disk modes. It has been shown that the vibration of a rotating disk is strongly coupled with both its ambient flow field and the acoustic field induced due to disk vibrations. Disk flutter is observed for some modes when rotational speed is above certain values.

The control forces are related to the velocity induced by the PZT actuator, which is proportional to the vibration displacement of the enclosure cover. Such vibration displacements generated by the actuator patches have been investigated under different configurations in terms of the number of patches, the area of individual patch and the spatial distribution between the patches. The configurations of PZT actuator patches affect flutter control performance through the dynamics of air flow that alter the control force matrix. Such control force matrix is evaluated through numerical analysis of the disk-enclosure-actuator system under certain parameters for four different cases, namely, one patch, two patches, three patches, and four patches. The results demonstrate that the control of flutter suppression can in general be achieved by either a single actuator patch or multiple patches. In case of one single actuator patch, a better control for all three flutter modes can be achieved when the sector angle is 360 degrees pasted in center. In case of two/three actuator patches, the optimization study shows that a better control performance to suppress all three flutter modes, especially for the high flutter modes, can be achieved by manipulating the relative locations of actuator patches and different control phases among them. Further studies on parameter optimizations such as the radius of PZT actuator as well as positions will be conducted and results will be reported in due time.

## Figures and Tables

**Table 1.** Geometric and material parameters of the hard disk platter.

**Parameter**	**Value**
Young’s modulus, *E*	71 (GPa)
Density of disk, *ρ_d_*	2.7 × 10^3^ (kg/m^3^)
Outer radius, *r_o_*	4.474 (cm)
Inner radius, *r_i_*	1.56 (cm)
Outer radius of the enclosure, *r_e_*	4.94 cm
Thickness, *h*	0.790 (mm)
Poisson’s ratio of the disk, ν	0.33
Density of air, *ρ_a_*	1.21 (kg/m^3^)
Speed of sound in air, *a*	343 (m/s)

**Table 2.** Comparisons of Numerical and Experimental Results (Rotating Speed Ω = 5,400 rpm).

**Mode**	**Analysis (Hz)**	**Experiments [17] (Hz)**	**Percentage Error %**
(0,0)	644	604	6.62%
(0,1)F	729	700	4.14%
(0,1)B	549	520	5.58%
(0,2)F	936	900	4.00%
(0,2)B	576	544	5.88%
(0,3)F	1,465	1,480	−1.01%
(0,3)B	925	892	3.70%

**Table 3.** The Control Effectiveness Generated by Passive Squeezing Patches.

	**Parameters**		
**Configuration**	**Distance to Disk Surface (mm)**	**Generated Pressure (atm)**	**Generated Maximum Speed (m/s)**
Case 1: Two Squeezing Plates	0.5	1.94	571
	1.0	1.66	533
	1.5	1.48	497
Case 2: Three Squeezing Plates	0.5	1.88	566
	1.0	1.79	542
	1.5	1.62	519
Case 3: Four Squeezing Plates	0.5	1.86	561
	1.0	1.73	540
	1.5	1.57	511

**Table 4.** Contour plots of Pressure and Velocity Generated by Passive Squeezing Patches.

Case	*d* = 0.5 mm	*d* = 1.0 mm	*d* = 1.5 mm
Case 1	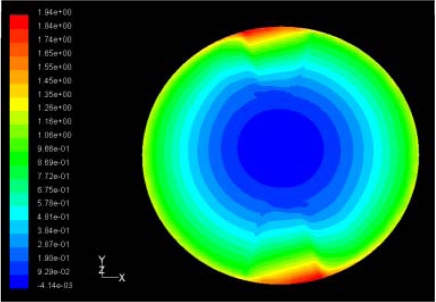 Max Pressure 1.94 (atm)	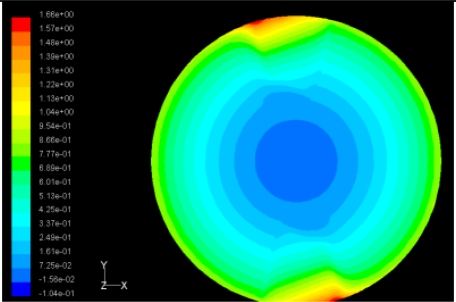 Max Pressure 1.66 (atm)	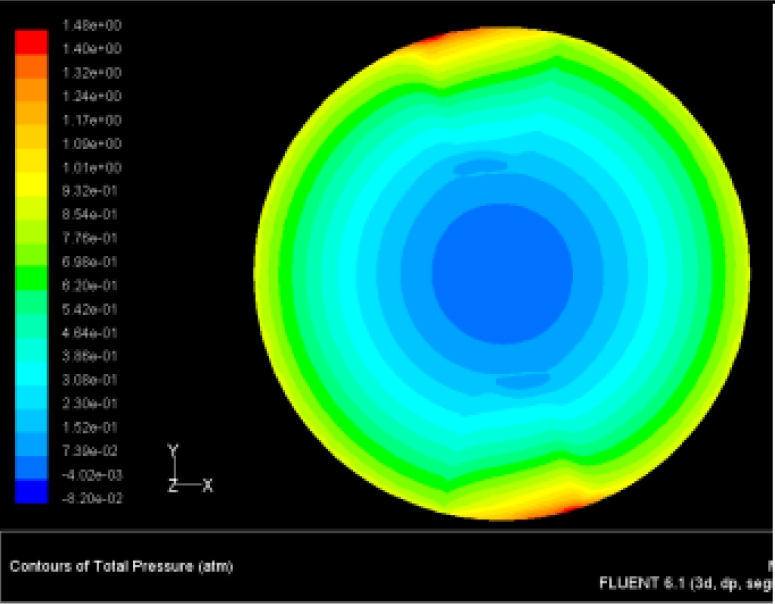 Max Pressure 1.48 (atm)
	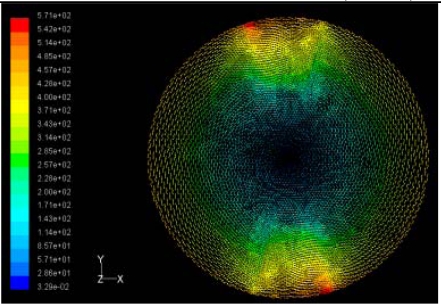 Max Speed: 571 m/s	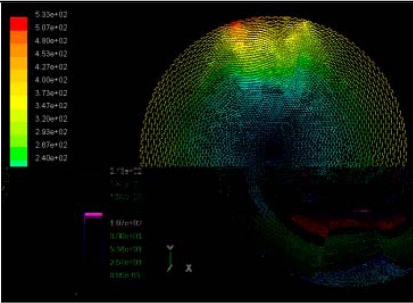 Max Speed: 533 m/s	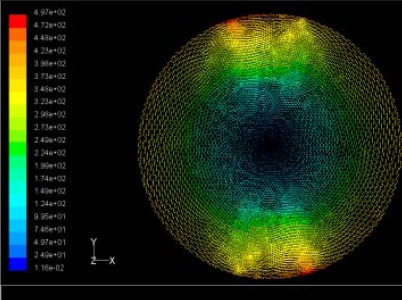 Max Speed: 497 m/s
Case 2	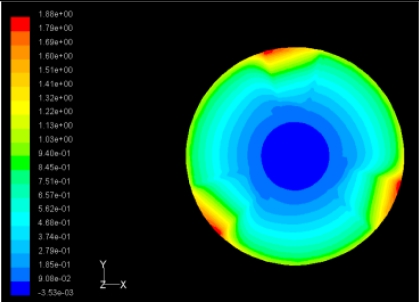 Max Pressure 1.88 (atm)	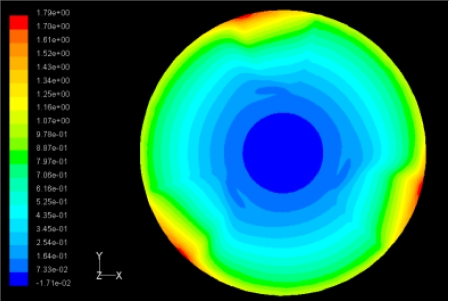 Max Pressure 1.79 (atm)	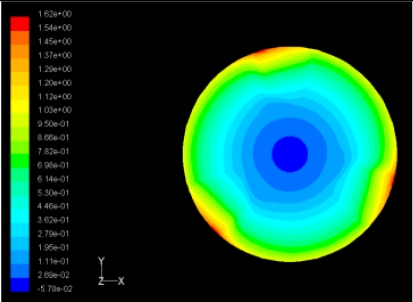 Max Pressure 1.62 (atm)
	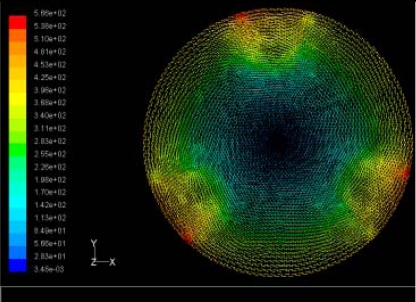 Max Speed: 566 m/s	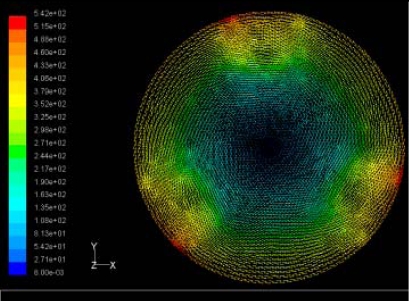 Max Speed: 542 m/s	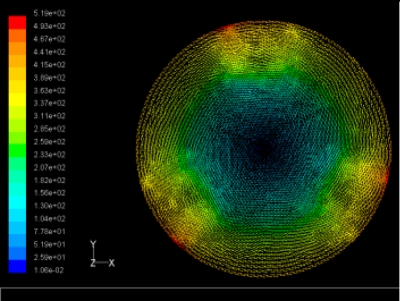 Max Speed: 519 m/s
Case 3	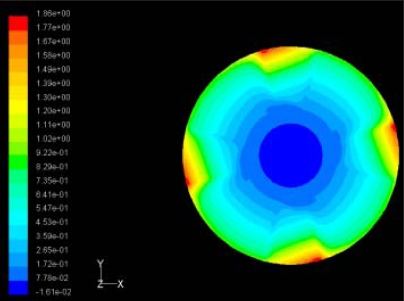 Max Pressure 1.86 (atm)	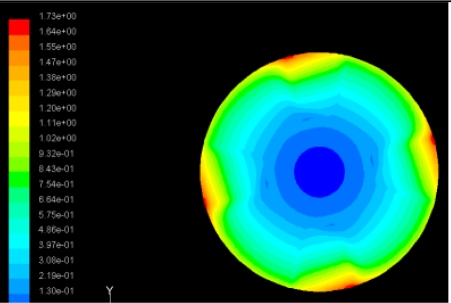 Max Pressure 1.73 (atm)	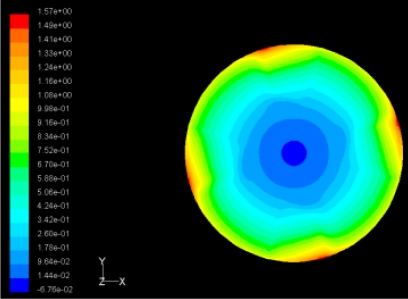 Max Pressure 1.57 (atm)
	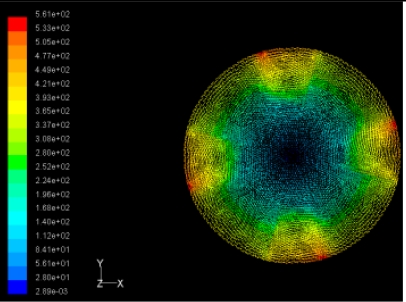 Max Speed: 561 m/s	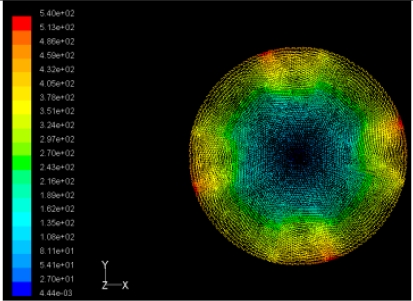 Max Speed: 540 m/s	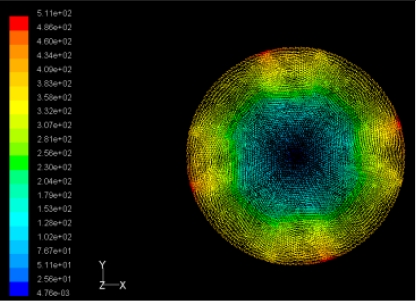 Max Speed: 511 m/s

**Table 5.** Contour Plots of Displacement Generated by Active PZT Patches on Encclosure.

Case 1 One PZT Patch	
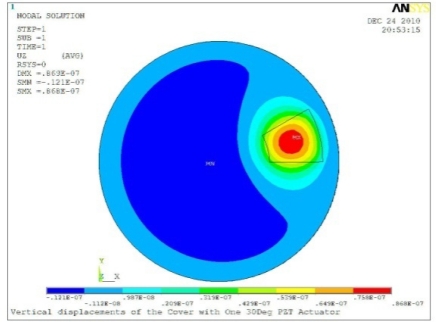 Δ*θ =* 30°, *Z_sum_* = 0.0868 μm	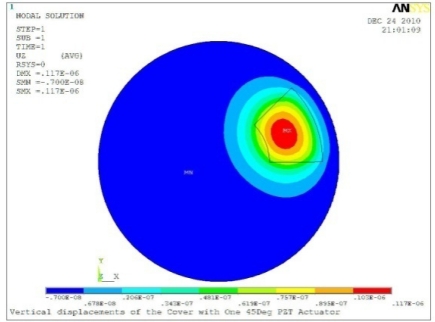 Δ*θ* = 45°, *Z_sum_* = 0.117 μm,
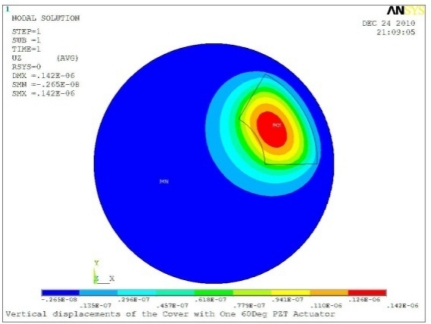 Δ*θ* = 60°, *Z_sum_* = 0.142 μm	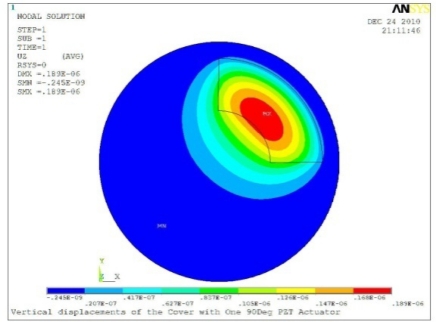 Δ*θ* = 90°, *Z_sum_* = 0.189 μm,
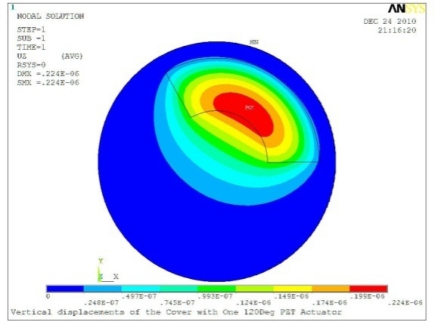 Δ*θ* = 120°, *Z_sum_* = 0.224 μm	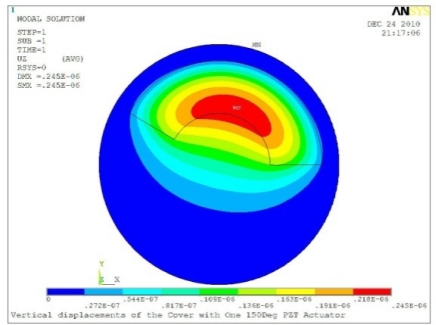 Δ*θ* = 150°, *Z_sum_* = 0.245 μm
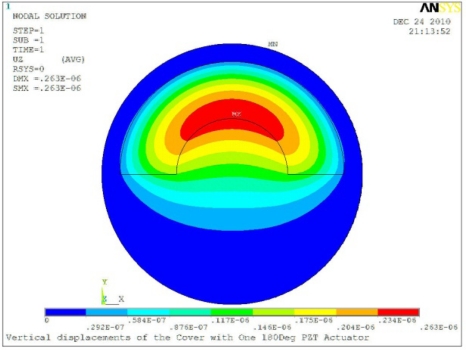 Δ*θ* = 180°, *Z_sum_* = 0.263 μm	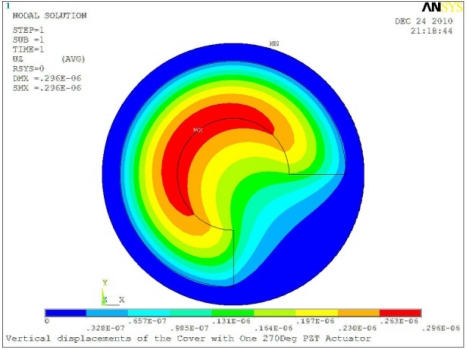 Δ*θ* = 270°, *Z_sum_* = 0.296 μm
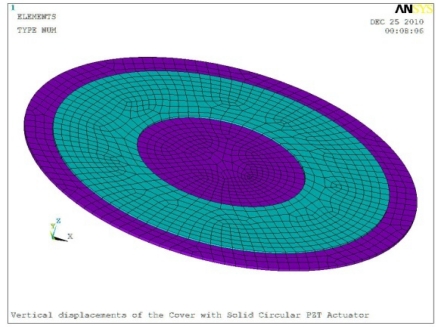 Δ*θ* = 360°	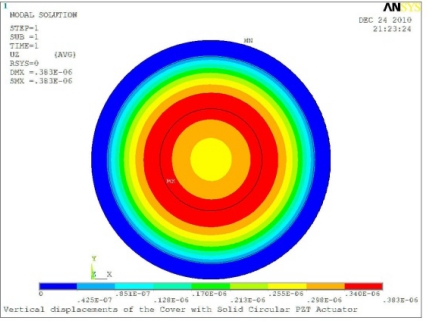 Δ*θ* = 360°, *Z_sum_* = 0.383 μm
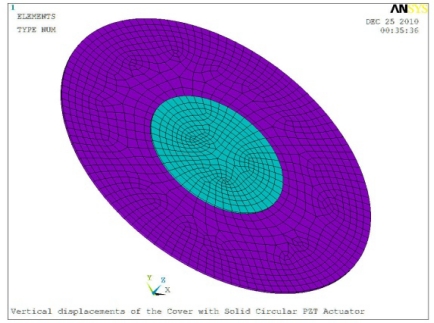 Δ*θ* = 360°, *r_0_* = 0.5 *r0, r_i_* = 0	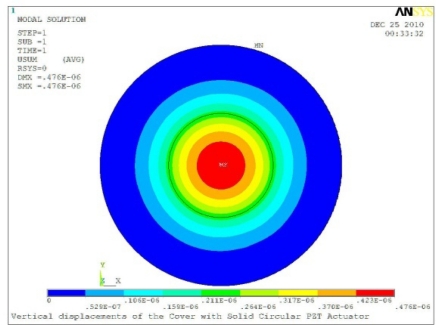 Δ*θ* = 360°, *Z_sum_* = 0.476 μm
Case 2 Two PZT Patches (*α* = 120°)	
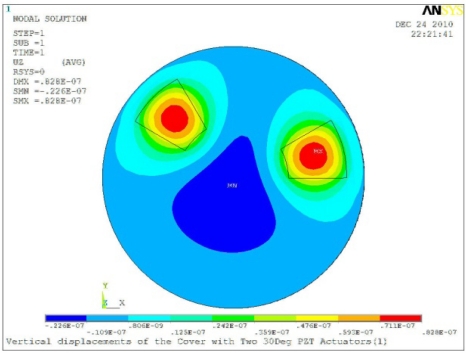 Δ*θ* = 30°, *Z_sum_* = 0.0828 μm	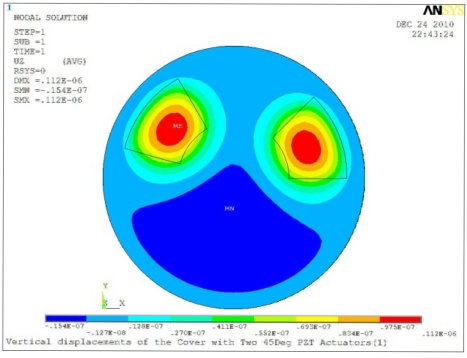 Δ*θ* = 45°, *Z_sum_* = 0.112 μm
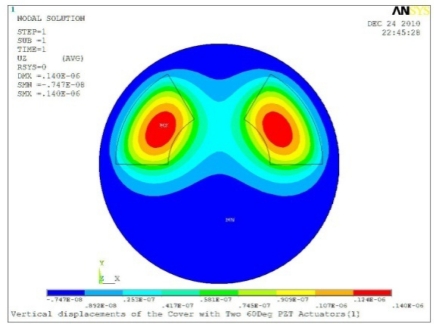 Δ*θ* = 60°, *Z_sum_* = 0.140 μm	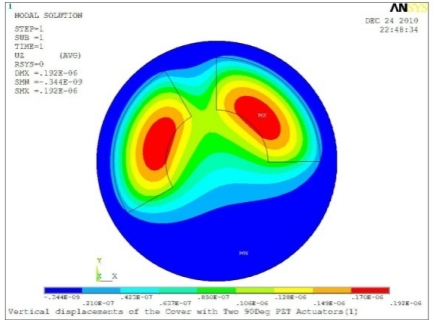 Δ*θ* = 90°, *Z_sum_* = 0.192 μm
Case 2 Two PZT Patches (*α* = 180°)	
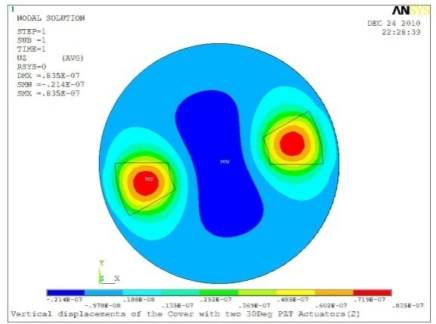 Δ*θ* = 30°, *Z_sum_* = 0.0835 μm	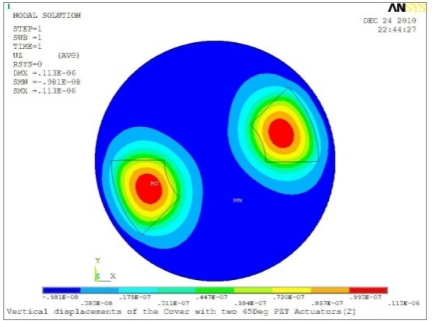 Δ*θ* = 45°, *Z_sum_* = 0.113 μm
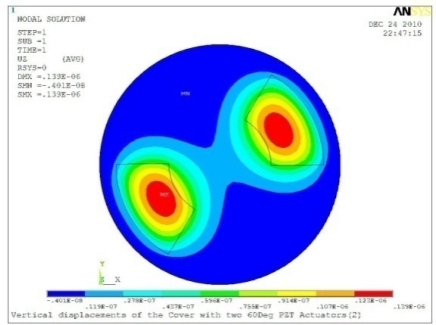 Δ*θ* = 60°, *Z_sum_* = 0.139 μm	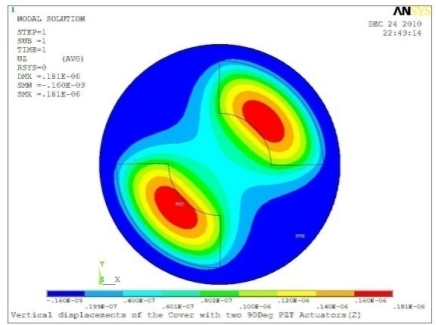 Δ*θ* = 90°, *Z_sum_* = 0.181 μm
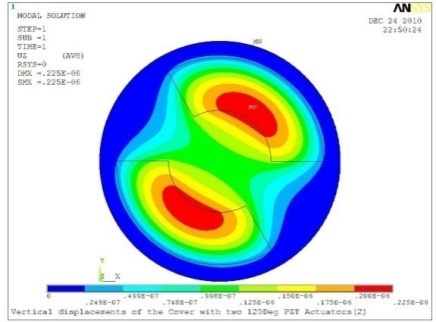 Δ*θ* = 120°, *Z_sum_* = 0.225 μm	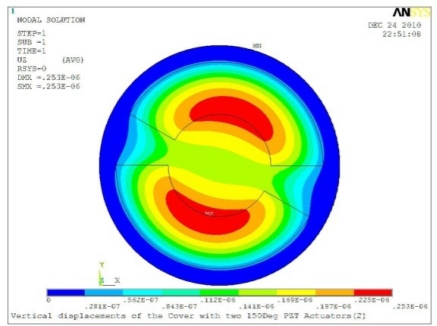 Δ*θ* = 150°, *Z_sum_* = 0.253 μm
Case 3 Three PZT Patches (*Δβ* = 120°)	
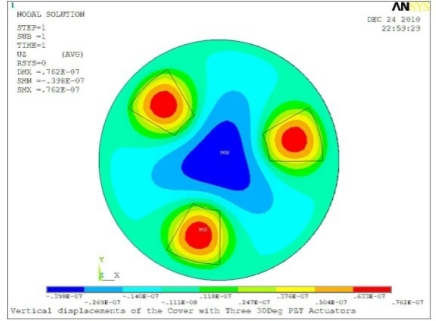 Δ*θ* = 30°, *Z_sum_* = 0.0762 μm	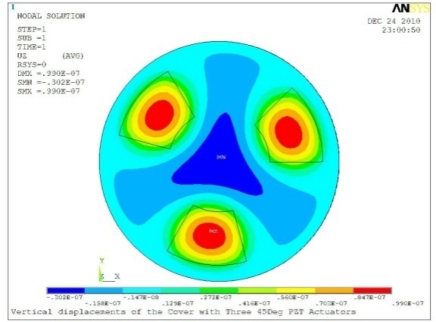 Δ*θ* = 45°, *Z_sum_* = 0.0990 μm
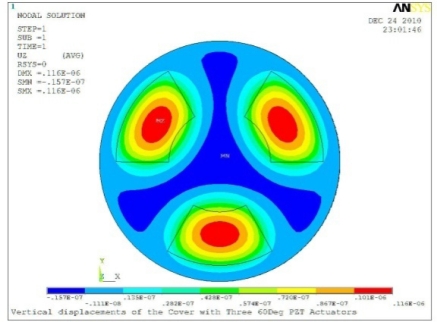 Δ*θ* = 60°, *Z_sum_* = 0.114 μm	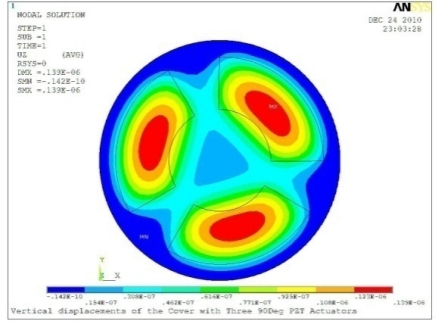 Δ*θ* = 90°, *Z_sum_* = 0.139 μm
Case 4 Four PZT Patches (*Δβ* = 90°)	
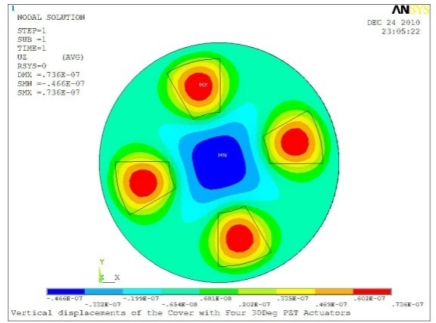 Δ*θ* = 30°, *Z_sum_* = 0.0736 μm	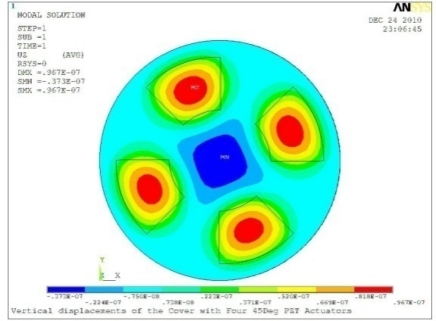 Δ*θ* = 45°, *Z_sum_* = 0.0967 μm
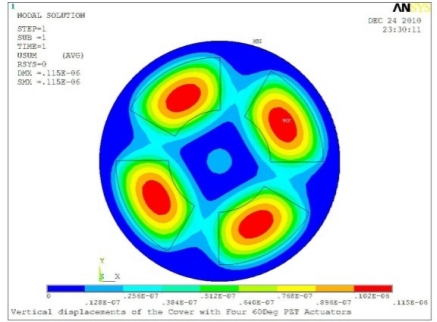 Δ*θ* = 60°, *Z_sum_* = 0.115 μm	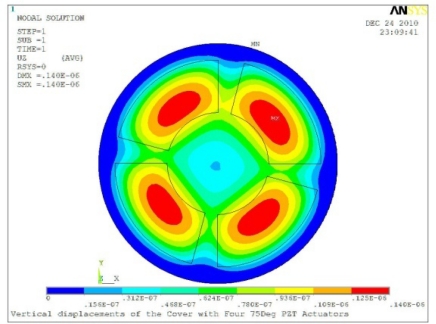 Δ*θ* = 75°, *Z_sum_* = 0.140 μm
